# Targeting the Hedgehog Pathway in Pediatric Medulloblastoma

**DOI:** 10.3390/cancers7040880

**Published:** 2015-10-23

**Authors:** Sherri Y. Huang, Jer-Yen Yang

**Affiliations:** 1 Department of Pharmacology and Toxicology, Indiana University School of Medicine, Indianapolis, IN 46202, USA; syhuangpcr@gmail.com; 2 Department of Basic Medical Sciences, College of Veterinary Medicine, Purdue University, West Lafayette, IN 47906, USA; 3 Center for Cancer Research, Purdue University, West Lafayette, IN 47906, USA

**Keywords:** hedgehog, Gli1, medulloblastoma

## Abstract

Medulloblastoma (MB), a primitive neuroectomal tumor of the cerebellum, is the most common malignant pediatric brain tumor. The cause of MB is largely unknown, but aberrant activation of Hedgehog (Hh) pathway is responsible for ~30% of MB. Despite aggressive treatment with surgical resection, radiation and chemotherapy, 70%–80% of pediatric medulloblastoma cases can be controlled, but most treated patients suffer devastating side effects. Therefore, developing a new effective treatment strategy is urgently needed. Hh signaling controls transcription of target genes by regulating activities of the three Glioma-associated oncogene (Gli1-3) transcription factors. In this review, we will focus on current clinical treatment options of MB and discuss mechanisms of drug resistance. In addition, we will describe current known molecular pathways which crosstalk with the Hedgehog pathway both in the context of medulloblastoma and non-medulloblastoma cancer development. Finally, we will introduce post-translational modifications that modulate Gli1 activity and summarize the positive and negative regulations of the Hh/Gli1 pathway. Towards developing novel combination therapies for medulloblastoma treatment, current information on interacting pathways and direct regulation of Hh signaling should prove critical.

## 1. Clinical Description of Medulloblastoma

Medulloblastoma (MB) is a rapidly-growing, highly invasive tumor arising from embryonal cells in the cerebellum. It is the most common malignant brain tumor in pediatric patients, accounting for about 20% of all brain tumors in children [1,2]. The majority of patients present clinically with subtle neurological symptoms that may persist for three months or more before diagnosis. These include headaches, vomiting, and lethargy as a result of increased intracranial pressure. Ataxia in the lower limbs as a result of cerebellar invasion may also occur. Brainstem involvement results in clinical signs such as cranial nerve defects, including Parinaud’s Syndrome (upward gaze and pupillary defect), facial weakness, ringing in the ears, and hearing loss [3,4]. Head tilt, from either trochlear nerve involvement or cerebellar tonsil herniation, can also occur [5,6].

The World Health Organization classifies medulloblastoma into subtypes based on histological traits: (1) desmoplastic or nodular; (2) medulloblastoma with extensive nodularity; (3) large-cell variant; and (4) anaplastic medulloblastoma [7]. However, disease prognosis is better predicted by four different non-histological subgroups, based on the global genome sequencing profile. Four principal transcriptional subgroups have been identified: (1) WNT; (2) Sonic hedgehog (SHH); (3) Group 3 (N-MYC amplification); and (4) Group 4 (heterogenous genes) [8,9]. Besides differing genome profiles, meta-analysis of clinical and molecular medulloblastoma data showed that these four subgroups also differ in terms of patient survival, DNA copy-number aberrations and demographics. WNT-MBs demonstrate the best survival outcome, with 95% 5- and 10-year overall survival in children and 100% five-year in adults, while Group 3 and Group 4 demonstrate the worst survival outcome, and this may be correlated with the higher frequency of 17p chromosome loss in these groups [10,11]. Out of these four, SHH-MBs are most common in infants and in adults, and small molecular inhibitors have been developed targeting SHH signaling [12].

However, as will be described in further detail in this review, current therapies targeting SHH signaling demonstrate several limitations. Towards exploring new venues of drug development for SHH signaling, this review will describe limitations with current therapies, pathways interacting with SHH signaling in MB, as well as pathways interacting with SHH in other cancer types. Finally, the review will discuss points of direct regulation of SHH signaling by different kinases and provide critical insight for drug-resistant in MB treatments.

## 2. Hedgehog Pathway

Hedgehog (Hh) signaling is mediated by a group of morphogen ligands: sonic hedgehog (SHH), Desert hedgehog (DHH) and Indian hedgehog (IHH). These are synthesized as precursor proteins that are then processed into two fragments, namely an amino-terminal peptide and a carboxy-terminal peptide. The amino-terminal peptide is responsible for Hh signaling [13,14,15]. Both the N- and C-termini of the amino-terminal Hh peptide are modified with lipid moieties, catalyzed in part by the carboxy-terminal peptide [16]. These lipid modifications must either be cleaved such that the secreted ligand is soluble or shielded in a transport mechanism through the bloodstream. In *Drosophila*, it has been shown that heparin sulfate glycoproteins called glypicans play a role in the transport of Hh ligand [17]. These glypicans can recruit lipophorins, lipoproteins that transport the hydrophobic Hh ligand through the bloodstream.

Hh signaling requires intact primary cilium, a microtubule-containing organelle that extends from the surface of nearly all cells in mammalian tissues. Responding to mechanical and chemosensation, primary cilium are localization points for signaling receptors, ion channels and transporters. Acting at the primary cilium, Hh morphogens play essential roles in embryogenesis, cell proliferation and tissue development, and stem cell maintenance [18]. Specifically at the activation of Hh signaling, Hh morphogens bind to the 12 pass-transmembrane receptor, PATCH (*PTCH1*) which is localized to the base of primary cilium, releasing its inhibition of Smoothened (*SMO*), a protein responsible for activating the downstream Hh pathway. Once SMO is activated, it binds to SUFU and induces nuclear translocation of Hh pathway transcription regulators, Gli1 (activator), Gli2 (activator) and Gli3 (repressor) [19,20]. Gli1, Gli2 and Gli3 regulate the expression of downstream targets such as *Gli1*, *Ptch1*, *cyclin D*, and *myc* involved in cell survival, proliferation and differentiation [21,22]. Later in this review, recent literature on direct regulation of the SHH signaling pathway will be discussed in the context of potential therapeutic development.

## 3. SHH in Medulloblastoma

During cerebellar development (postnatal day 3 to day 9), granule neuron progenitor (GNPs) cells in the outermost layer of the cerebellum (termed the external granule layer or EGL) undergo massive proliferation through SHH signaling [23]. In one study, blocking SHH signaling through anti-SHH antibodies in chick cerebellum led to a marked decrease in cerebellar size [24]. Thus, SHH signaling is critical for normal cerebellar development. However, constitutive activation of SHH signaling results in tumorigenesis. *Smoothened* mutations were detected in sporadic basal cell carcinomas and primary neuroectodermal tumors, and activating mutations in *Smoothened* lead to cerebellar dysplasia and tumors [25,26,27]. In one study, 50% of transgenic mice expressing one copy of constitutively active *Smoothened* developed medulloblastoma-like cerebellar tumors by five months of age; this incidence increased to over 80% in mice homozygous for constitutively active *Smoothened*. Mice harboring homozygous constitutively active *Smoothened* also demonstrated metastasis of cerebellar tumors to the leptomeningeal membranes of the brain [28]. Other components of the SHH pathway, upon mutation, are sufficient to drive medulloblastoma development. Mutations in *PTCH* were found in a subset of sporadic medulloblastomas [29]. In addition, deletion of just one copy of *PTCH* is sufficient to drive medulloblastoma, as the majority of *PTCH* hemizygous mice with medulloblastoma was found to contain the wild-type sequence in the second *PTCH* allele [30]. A subset of pediatric medulloblastoma patients harbor mutations, either germline or somatic, in *SUFU* [31].

Activity of downstream Gli transcriptional activity is closely linked with tumorigenesis. In one study, inactivation of both *Gli1* alleles resulted in a decrease in spontaneous medulloblastoma formation in PTCH heterozygote mice [32]. However, it has also been suggested that SHH signaling can direct tumorigenesis in a transcriptionally-independent manner. In an experiment, Transwell migration experiments with mesenchymal fibroblasts were performed in the presence and absence of transcriptional inhibitor actinomycin D, which abolished Gli-reporter activity. However, migration was not inhibited, suggesting that SHH signaling may mediate cancer-related phenotypes such as mesenchymal migration through Gli-independent mechanisms. Indeed the same study found that SHH-mediated migration is accomplished through leukotriene synthesis, suggesting a combination therapy target of both SHH and leukotriene synthesis pathways for cancer types [33]. The relevance of these studies must be assessed in the context of medulloblastoma.

A recent genome sequencing study suggests that infant (≤3 years old), pediatric (4–17 years old) and adult SHH-medulloblastomas are genetically distinct. Sequencing of infant, children and adult medulloblastomas found *PTCH1* mutations in approximately equal frequencies across all age groups. On the other hand, *SUFU* mutations were found enriched in infant SHH-MB, while *SMO* mutations were enriched in adults. Mutations in TP53 were found in nearly 50% of the 4–17-year-old age group samples [34]. These results suggest that treatment of medulloblastoma should consider the unique genetic backgrounds of infant, pediatric and adult SHH-MBs.

For medulloblastoma, the first treatment option is usually surgery to resect as much of the tumor as possible. This is followed by radiation of the brain and spine and/or chemotherapy [35]. Standard chemotherapy regimen for medulloblastoma have historically largely included alkylating agents such as cisplatin, vincristine, carboplatin, cyclophosphamide and lomustine. Side effects of current chemotherapy treatment for medulloblastoma include endocrine perturbations, shortened height in the patient, neurocognitive impairments, secondary malignancies, hearing loss, and cardiopulmonary problems [36,37]. Other current treatment options largely target SMO but demonstrate limitations such as drug resistance due to SMO mutations which we will discuss it later.

Use of the steroidal alkaloid cyclopamine, which targets SMO to suppress Hh signaling, launched the idea of modulating the Hh signaling pathway as a therapeutic [38,39,40]. Cyclopamine is found to suppress tumor growth in a variety of cancers and is used for studying the regulation of Hh signaling extensively. Irregular activation of the hedgehog-signaling pathway is the critical abnormality compelling the growth of basal cell carcinomas (BCCs), and chronic oral administration of cyclopamine noticeably diminishes 66% UVB induced BCC formation in Ptch1^+/−^ mice [41]. Cyclopamine also suppresses medulloblastoma development in Ptch1^+/−^ mice [42]. Unfortunately, cyclopamine is not proper for clinical development because of its low oral solubility [43].

More SMO inhibitors are currently being evaluated, including vismodegib (GDC-0449), sonidegib (LDE-225), BMS-833923, PF-04449913 and LY2940680, in clinical trials in advanced cancers [44]. Among these, vismodegib became the first U.S. Food and Drug Administration (FDA) approved SMO antagonist for the treatment of advanced or metastatic BCC in 2012 [45]. Vismodegib significantly lessens the rate of appearance of new BCCs in patients [46]. Nonetheless, reports showed that most BCCs regrow after vismodegib is stopped [47]. In addition, a phase II trial to estimate vismodegib efficacy in ovarian cancer patients showed that vismodegib did not meet the primary endpoint, and only a modest enhancement in progression free survival was detected for vismodegib compared to placebo (7.5 *vs.* 5.8 months) [48]. Likewise, a phase II study of vismodegib in patients with advanced chondrosarcoma did not meet the primary endpoint [49]. Finally, in a Phase I study of vismodegib in pediatric patients, out of three patients with SHH subtype medulloblastoma treated with vismodegib, only one patient demonstrated an antitumor response [50].

GANT61 and HPI-1 are potential therapeutics for MB that target the Hh signaling pathway; both target Gli1 and Gli2 [51,52,53]. LDE-225, BMS-833923 and saridegib target SMO [54,55,56]. While these therapeutics target Hh signaling-mediated cancers, they have not been tested in the context of SHH-MB (Table 1).

**Table 1:** Current medulloblastoma therapeutics and Hh-pathway targets that potentially may be applied to SHH-MB.

Current and Potential Pipeline Medulloblastoma Drugs			
Drug	Target	Pathway Affected	Limitations
**Alkylating agents**	DNA	DNA repair	Range of side effects
**Cyclopamine**	SMO	Hh	Low oral solubility
**Vismodegib (GDC-0449)**	SMO	Hh	Possible limited efficacy; mutations in SMO contributes to resistance
**LDE-225**	SMO	Hh	In mouse model, tumors originally shrunk but then regrew [54]
**GANT61 [52]**	Gli1 and Gli2	Hh	Tested in prostate cancer cells; Remains to be tested with medulloblastoma
**HPI-1 [53]**	Gli1 and Gli2	Hh	Preclinical, remains to be tested with medulloblastoma
**BMS-833923 [55]**	SMO	Hh	Clinical trials with gastric and esophageal cancers; remains to be tested with medulloblastoma
**Saridegib (IPI-926) [56]**	SMO	Hh	Preclinical

In the context of SHH-MB, Kool *et al.* 2014 argue that patients with mutations downstream of *SMO* such as those with *GLI2* and *NMYC* mutations generally would not respond to *SMO* antagonists. Patients with genetic aberrations upstream of *SMO*, such as *PTCH1* and *SHH* amplifications, respond to *SMO* antagonists and include pediatric patients. Therefore, exploration of *SMO* inhibitors in the treatment of pediatric medulloblastoma is productive, especially when used in combination therapies. Identification of pathways cross-talking with SHH, along with an understanding of pathways to treatment resistance, will aid the development of more efficacious SHH targeting therapeutics.

## 4. Genes Linked to Treat Hedgehog Inhibitors Resistance

A number of genes have been linked to treatment resistance. In one study, medulloblastoma cells surviving radiation were analyzed for the expression of genes for stem cell behavior, which is linked to the ability of a subset of tumor cells to drive tumor formation or initiate metastasis. *ABCG2*, an ATP-binding cassette (ABC) transporter associated with stem cell behavior and drug resistance, was found to be elevated. Treatment of surviving cells from certain patients with ABC transporter inhibitors verapamil and reserpine sensitized the cells to radiation [57]. High levels of ABCG2 are a property of stem cells, and ABC transporters are purported to efflux substances toxic to cells, which potentially include chemotherapeutic agents [58]. Thus, it is not surprising that ABC transporters are associated with radiation treatment resistance. Furthermore, as the study only assessed stem cell behavior genes, additional studies on the differential gene expression of cells responsive to and resistant to radiation may reveal more gene candidates responsible for resistance.

The role of cancer testis antigens (CTA), which are highly elevated across different cancers including melanoma and breast cancer, in modulating chemotherapy resistance has been investigated in the context of medulloblastoma. Expression of members of the CTA family, such as the MAGE and GAGE proteins, were correlated with chemotherapy resistance, and inhibition of these genes sensitized MB cells to agents such as cisplatin and etoposide [59].

Acquired mutations in components of the SHH pathway will also drive resistance to therapies targeting SHH signaling. In a patient with metastatic medulloblastoma, an acquired G-to-C missense mutation at position 1697 (aspartic acid to histidine) in *Smoothened* was discovered to contribute to resistance to the Smo-targeting drug GDC-0449, through a deficiency in drug binding [60]. Whole exome sequencing of basal cell carcinomas in Gorlin Syndrome patients resistant to vismodegib treatment revealed acquired mutations in either the drug-binding pocket or outside this region. The authors suggest that mutations in the drug-binding pocket may alter binding of an endogenous ligand. Mutations outside the drug-binding pocket in general increased SHH signaling basal activity, possibly mediated through a *PTCH1* mutation [61].

## 5. Considerations for Targeting the SHH Pathway in MB

In light of recent literature, several considerations are important for future therapeutic development for SHH medulloblastoma. The first consideration is the variable genetic backgrounds of SHH-MB in infants, children and adults. For example, patients with mutations upstream in the SHH pathway (*SHH* amplifications, *PTCH1* and *SMO* mutations) in general may respond more favorably to SMO inhibitors, while those with mutations downstream (*GLI2* mutations) may be more resistant to treatment [34]. The second is the development of resistance. An understanding of the radiation and chemotherapy resistance mechanisms of medulloblastoma is necessary for efficient targeting of medulloblastoma. Assessing the influence of crosstalk from other signaling pathways will significantly contribute to novel therapeutics for MB with regard to combination therapeutics. This review assesses the role of other signaling pathways implicated in SHH-MB and pathways which act synergistically with SHH signaling to promote tumorigenesis with other cancers; these latter pathways represent points for further investigation in the context of SHH-MB. Finally, we will discuss points of direct regulation of components in the SHH signaling pathway.

## 6. Pathways Interacting with SHH Signaling in MB

This section will discuss pathways interacting with SHH signaling. Components of these pathways may represent venues for further investigation, especially in the context of combination therapy with the SHH pathway.

**p53.** In light of the observation from Kool *et al.* 2014 that TP53 was enriched in pediatric (ages 4–17) MBs, the role of p53 in MB should be examined closely. p53 status is important in the prognosis of patients with SHH-medulloblastoma, as well as disease incidence. A cohort study suggested that TP53 status plays a critical role in survival status of patients, with five-year survival rates differing significantly between 41% and 81%, respectively, for SHH-medulloblastoma patients with and without TP53 mutations [62]. In mice with one deletion of *PTCH*, incidence of medulloblastoma was 14%; this incidence increases to >95% in the presence of p53 loss, and this increase is specific for p53 loss. However, no significant change in incidence was observed for heterozygous mice carrying a mutation in either APC or p19ARF [63]. Finally, reduction of levels of MDM2, a negative regulator of p53, resulted in decreased expression of Gli1 and Gli2 along with small cerebella characteristic of reduced SHH signaling. In addition, SHH signaling in granule neuronal precursors promoted MDM2 accumulation and MDM2 reduction prevented tumorigenesis in PTCH heterozygous mutant mice [64]. These results suggest that MDM2 interacts closely with SHH signaling in promoting medulloblastoma and poses as an attractive candidate for co-inhibition with SHH signaling in the treatment of medulloblastoma. Other modulators of p53 activity should also be further studied for their potential role in SHH-MB combination therapy.

**cAMP.** The role of cAMP in a variety of brain tumors has been established. Furman *et al.* found that higher cAMP levels correlated with lower grade brain tumors, while lower adenylate cyclase and cAMP levels correlated with higher malignancy [65]. In a neurofibromatosis-1 model, Warrington *et al.* generated foci of decreased cAMP using phosphodiesterase-4A1 in mice cortex and found the majority of mice developed cortical growth with tumor morphology [66]. Possibly linking cAMP regulation to SHH-mediated MB, Pan *et al.* found that the phosphorylation by cAMP-dependent PKA results in Gli2 degradation [67]. These results suggest that cAMP is an important coupler of upstream receptors to SHH signaling.

Further linking cAMP to SHH-mediated MB, another group of researchers found that ablation of the *GNAS* gene, encoding the G protein Gαs, is sufficient to initiate SHH medulloblastoma. Downstream Gαs signaling mediated through cAMP levels suppresses SHH signaling, and mice harboring the *GNAS* mutation demonstrate decreased tumor proliferation when cAMP levels are elevated [68]. Therefore, Gαs is another attractive candidate for co-inhibition with SHH signaling.

**Atoh1 and Boc.** Defects in different components of Hh signaling during cerebellar development trigger formation of medulloblastoma. For example, following SHH-induced proliferation, the GNPs exit the cell cycle and migrate inwards the cerebellum where they differentiate into granule neurons in the internal granule layer (IGL). Important in this process is the transcription factor Atoh1, whose expression is restricted to proliferating GNPs and absent in IGL neurons. SHH signaling appears to stabilize Atoh1 protein levels by preventing its proteasomal degradation through a phosphor-dependent mechanism. Abnormal constitutive SHH signaling may lead to Atoh1 overexpression, subsequently transforming GNPs into MB cells [69]. Another study found that the SHH-binding protein Boc is upregulated in medulloblastomas. In addition, Boc inactivation resulted in reduced proliferation and progression of early medulloblastomas to advanced cancer [70]. Both Ato1 and Boc represent potential combination therapy targets.

**TGF-β.** Microarray analyses of medulloblastoma samples from patients with primary and recurrent and metastatic tumors was performed, and TGF-β was found to be significantly differentially expressed. With primary tumors, positive nuclear staining of SMAD3, representing activation of TGF-β pathway, correlated with longer survival [71]. In contrast with these findings, however, another group assessed the effect of T-cell TGF-β signaling on MB progression. Abrogation of T-cell TGF-β signaling in mice harboring a dominant-negative form of TGF-β receptor and an activating Smoothened mutation mitigated MB progression [72]. Therefore, the role of TGF-β signaling and Smad3 activation in survival or progression of MB and should be furthered studied.

Bone morphogenic proteins, which belong to the TGF-β superfamily of morphogens, play a role in controlling SHH-mediated cell proliferation. Specifically, BMP2 and BMP4 have been shown in primary culture of proliferating granule cell precursors to arrest SHH-induced proliferation, thus allowing the cellular differentiation. Moreover, upon addition of BMP2 to SHH-stimulated cultures, *Smo* and *Gli1* mRNA expression were downregulated while *Ptch* was unaffected. Finally, overexpression of Smad5 was shown to decrease the percentage of SHH-induced proliferating cells in culture. These results suggest that the BMP2-Smad5 axis, specifically its activation, may be an important targeting point that allow a bypass to SHH-induced proliferation [73].

**CXC12 and CXCR4.** The chemokine CXC12 and its receptor CXCR4 play a synergistic role with SHH signaling in medulloblastoma. CXC12 plays an important role during granular cell development. Deletion of CXCR4 or CXC12 resulted in migration of granule cell precursors away from the EGL [74]. In addition, it was shown that CXCL12 can induce granule cell chemotaxis. The observation that CXC12 is secreted from the pia surrounding the ECL led to a model whereby pia-secreted CXC12 promotes granule cell migration towards the EGL through chemotactic motion towards the pia [75]. Co-activation of both SHH and CXCR4 resulted in higher expression of *cyclin D1*, while CXCR4 antagonism, resulted in decreased *cyclin D1* expression and also blocked maximal MB tumor growth. Finally, SHH activation was shown to trigger CXCR4 localization to the cell surface [76]. These results suggest a close synergism between SHH and CXCR4 signaling in promoting granule cell proliferation leading to MB development. Therefore, the CXCR4 axis is an enticing point of inhibitory drug targeting.

**bFGF.** Basic FGF (bFGF) signaling appears to have an inhibitory role on SHH-induced proliferation. Indeed, co-incubation of SHH with bFGF eliminated the Shh-induced proliferation [77]. Consistent with these results, another research group found that treatment of MB cells with bFGF precluded tumor formation following transplantation of these cells and that injection of bFGF into tumors inhibited growth in mice [78]. In addition, bFGF inhibited expression of SHH pathway transcriptional targets *Gli1*, *Nmyc*, and *cyclin D1* [77]. Therefore, bFGF prevents SHH-mediated proliferation by interfering with Shh signaling upstream of *Gli1* transcription. Activation of bFGF is thus another attractive point of co-targeting in SHH-induced MB.

**Survivin.** Survivin regulates cell cycle progression and inhibits apoptosis [79]; Brun and collaborators revealed an important role for survivin in MB tumor proliferation and progression. The study determined that in PTCH-mutant MB cells, survivin expression was overexpressed. Isolated MB tumor cells from survivin-deleted mice demonstrated significantly decreased thymidine incorporation along with cell cycle arrest in the G2/M phase [80]. How survivin interacts with SHH signaling prompts further investigation, and survivin may represent another co-inhibition target.

## 7. Non-MB Tumors: Pathway Crosstalk with SHH

The crosstalk between RAS/RAF/MEK and Hh signaling has been demonstrated in several cancer models. In melanoma cells, oncogenic Ras activity, dominant active MEK1 and dominant active AKT1 all increased the subcellular nuclear localization of exogenous Gli1 [81]. Similarly, it was shown in pancreatic ductal adenocarcinomas (PDA) that cell lines expressing increased Ras protein drove an increase in Gli1 luciferase activity. This effect is mediated through the RAF/MEK/MAPK pathway, as MEK inhibition decreased the Gli1 luciferase activity whereas PI3K inhibition had no effect [82]. Specifically, Hh may require Ras signaling to drive the formation of pancreatic intraepithelial neoplasias (PIN), the earliest stage of PDA, as simultaneous activation of the two pathways drove PIN formation while Hh signaling initiated undifferentiated carcinomas [83]. Finally, using a hybrid computational search algorithm, Whisenant *et al.* identified that Gli1 and Gli3 were MAPK substrates. They can be phosphorylated by JNK1/2 and ERK2, these results provided a direct connection between MAP kinase and hedgehog signaling [84].

A growing number of studies demonstrate the influence of other signaling pathways on Hh signaling in breast cancers. For example, one study demonstrated cooperative regulation at the transcriptional and epigenetic level leading to SHH overexpression [85]. The authors of the study had shown previously that hypomethylation at the SHH promoter along with NF-kB activation resulted in SHH overexpression. They then show that possibly the promoter hypomethylation allowed access for NF-kB binding to induce SHH transcription. One immunohistochemical study found significant correlation between nuclear Gli1 and transcription factor FOXC2 expression in breast cancer tissues. This correlation associated with tissues with basal-like breast cancer phenotype and shorter survival times [86]. The p53 family of transcription factors have also been implicated with Hh signaling in breast cancer development. A member of this family, p63, which is a master regulator of epithelial stem cell maintenance, binds directly to transcriptional regulatory regions of sonic hedgehog pathway components, including SHH, Gli2 and PATCH genes. Knock-down of p63 was shown to restrict expansion and self-renewal of mammary cancer stem cells and delay tumor growth. This study thus linked the p63-mediated self-renewal of mammary stem cells with the SHH pathway by demonstrating a direct modulation of SHH genes by p63 [87].

The balance between cellular apoptosis and autophagy may be crucial in certain SHH-mediated tumors. This balance was found important for hepatocellular carcinoma (HCC) pathology and is mediated in part by Hh signaling. Treatment of cultured human hepatocellular carcinoma tissue with recombinant SHH or other Hh pathway agonists such as purmorphamine prevented the activation of autophagy. Inhibition of Hh signaling with GANT61, an inhibitor of Gli1 and Gli2, induced autophagy. This induction was associated with upregulation of Bnip3, a protein which mediates displacement of anti-apoptotic protein Bcl-2 from autophagy protein beclin-1. As Bcl-2 inhibits beclin-1 mediated autophagy, the inhibition of Hh signaling and subsequent upregulation of Bnip3 leads to upregulation of apoptosis over autophagy. Indeed, the study found that the inhibition of Hh signaling increased HCC apoptosis and decreased cell viability, suggesting that the balance between apoptosis and autophagy as mediated by Hh signaling is important in HCC pathology [88].

Additionally, in hepatocellular carcinoma, protein kinase C delta (PKCδ), which generally slows proliferation and induces cell cycle arrest of various cell lines, is found to suppress Hh signaling. PKCδ prevents GLI1 nuclear localization and inhibits GLI1 transcriptional activity, leading to suppression of Hh signaling [89].

A number of studies implicate the role of glucose metabolism and energy-sensing and Hh pathway in progressing HCC. Overexpression of pyruvate kinase isoenzyme 2 (PKM2) in HCC cells resulted in upregulation of Gli1 transcription, while shRNA-mediated downregulation of PKM2 resulted in downregulation of Gli1 transcription. Notably, PKM2 in tumor cells determines the fate of glucose use, *i.e.*, catabolic *vs.* anabolic reactions. The crosstalk between PKM2 and Hh signaling suggests an overlap of glucose metabolism with the Hh pathway [90]. Finally, the role of cellular energy homeostasis and Hh signaling in HCC development was also implicated in the study by Liu *et al.*, 2014. Overexpression of AMP kinase led to a decrease in Gli1 expression, while the knockdown of AMPK increased Gli1 expression [91]. Suppression of Hh signaling through downregulation of Gli1 may serve as another venue of targeting SHH-MBs. Direct regulation of SHH signaling, especially in the context of Gli1 regulation, will be discussed next.

## 8. Direct Regulation of SHH Signaling

The activity of Gli1 is crucial determined by its protein stability and localization. Several kinases were known to modulate Gli1 protein stability and localization. Protein kinase A (PKA) retains Gli1 in the cytoplasm and suppresses Gli1 transcriptional activity by phosphorylating it on Thr-374 [92]. In addition, PKA also phosphorylates Gli1 on Ser-640; this inhibits Hh signaling by promoting the Gli1/14-3-3 interaction [93].

Ribosomal protein S6 kinase 1 (S6K1) phosphorylates Gli1 on Ser-84, leading to GLI1 activation [94]. PI3K/AKT or RAS/RAF/MEK signaling is shown to crosstalk with Hh signaling. Treatment of melanoma cells with AKT1 inhibitors decreased the number of cells with nuclear Gli1 labeling, while increasing the number of cells with cytoplasmic Gli1 labeling, suggesting that AKT1 may be required for Gli1 nuclear localization and transcriptional activity [81]. However, as shown in neuroblastoma, Gli1 activity is suppressed by AKT2 which phosphorylates GSK3β, leading to GSK3β stabilizing the inhibitory SUFU/GLI1 complex [95].

**Figure 1:**
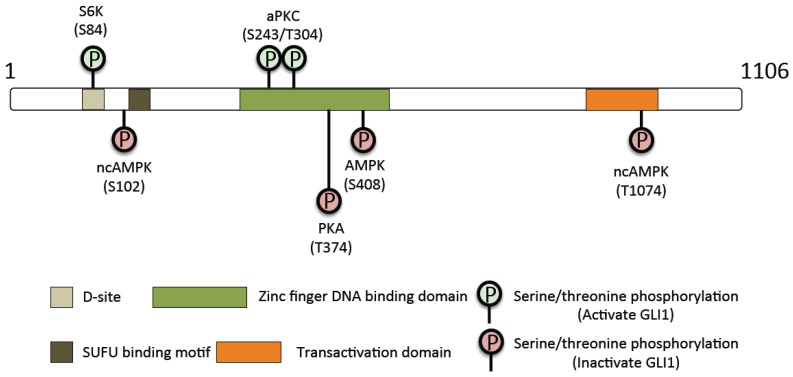
Summarize the specific phosphorylation sites on human GLI1.

Previously we found that activation of AMPK reduces Gli1 protein levels and stability, thus blocking SHH-induced transcriptional activity. AMPK phosphorylates Gli1 at Ser-102 and 408 and Thr-1074. Mutation of these three sites into alanine prevents phosphorylation by AMPK. This in turn leads to increased Gli1 protein stability, transcriptional activity, and oncogenic potency [96]. The kinases and phosphorylation sites that regulate Gli1 activity are summarized in Table 2 and in Figure 1. As Gli1 represents downstream SHH signaling, it is an attractive target for specific SHH mediated MB. Inhibition of its activating kinases or activation of its inhibitory kinases can be explored.

**Table 2:** GLI1 regulatory kinases and their sites of phosphorylation on GLI1.

GLI1 Regulatory Kinases		
Kinase	Residue	Activating or Inhibitory
Protein Kinase A	Thr-374	Inhibitory
Protein Kinase A	Ser-640	Inhibitory
Ribosomal protein S6 kinase 1	Ser-84	Activating
Atypical Protein Kinase C	Ser-243	Activating
Atypical Protein Kinase C	Thr-304	Activating
AMPK	Ser-102	Inhibitory
AMPK	Ser-408	Inhibitory
AMPK	Thr-1074	Inhibitory

## 9. Conclusions

Recent literature on the genetic diversity of MB suggests that adult, pediatric and infant MB have unique genetic signatures. Towards developing novel therapeutics for pediatric MB, an understanding of these unique genetic backgrounds is crucial. In addition, successful treatment of SHH MB will benefit from an understanding and targeting of downstream SHH signaling and pathways that cross-talk with SHH. To summarize, venues for continuing and further research include:

(1) Clinically, the identification and assessment of novel Gli inhibitors for Hh-mediated cancers should be evaluated in the context of medulloblastoma.
(2) Clinically, given the unique genetic backgrounds of adult *versus* pediatric MB, assessment of novel MB treatments should be conducted with stratification of patient groups in terms of age.
(3) Understanding of the resistance that arises from treatment of Hh-mediated cancers should be assessed in the context of Hh-mediated MB.
(4) Non-transcriptional mechanisms by which SHH signaling mediates tumorigenesis in MB should be further studied.
(5) Pathways such as the RAS/RAF/MEK, NF-κB, autophagy, and glucose sensing signaling have been shown to modulate SHH signaling. The effects of the crosstalk of these pathways on MB tumorigenesis should be further studied.
(6) Gli1 represents a downstream signaling point of SHH driving SHH-mediated MB, and knowledge about the regulation points on Gli1 will prove essential to developing therapeutics targeting this transcription factor.
